# Genome-based taxonomic classification of *Listeria* phage and diversity analysis of major capsid protein, receptor-binding protein and endolysin

**DOI:** 10.3389/fmicb.2026.1767683

**Published:** 2026-03-04

**Authors:** Jinni Chen, Hao Zhou, Lingyun Liu, Pan Mao, Lingling Li, Xuefang Xu, Ji Pu, Jing Yang, Hui Sun, Xia Luo, Yan Wang, Changyun Ye

**Affiliations:** National Key Laboratory of Intelligent Tracking and Forecasting for Infectious Diseases, National Institute for Communicable Disease Control and Prevention, Chinese Center for Disease Control and Prevention, Beijing, China

**Keywords:** *Listeria monocytogenes*, *Listeria* phage, MCP, RBP, endolysin

## Abstract

**Introduction:**

*Listeria monocytogenes* (Lm) poses a significant risk to food safety due to its adaptability and pathogenicity. In contrast, *Listeria* phages show great promise as biocontrol agents.

**Method:**

This study comprehensively analyzed 97 complete *Listeria* phage genomes from 14 countries across four continents, including 16 newly isolated phages exhibiting specific phenotypic characteristics.

**Results:**

The phages were grouped into nine genomic clusters that clearly distinguished between virulent and temperate lifestyles. Temperate phages demonstrated greater genomic diversity than virulent ones. Cluster 1 phages were assigned to the genus *Pecentumvirus*, exhibiting a broad geographical distribution with diverse sources, and appear to have an ecological advantage based on their genomic characteristics. Evolutionary analyses classified the major capsid protein (MCP), receptor-binding protein (RBP), and endolysin of *Listeria* phages into nine, seven, and eight distinct types, respectively. These three proteins exhibited high levels of conservation within the virulent clusters, but significant diversity within the temperate clusters. Notably, the RBPs of types R1, R2, R4, and R6 are associated with broad host ranges and distributed across Clusters 1, 2, 7, and 8 phages. Cluster 3 phages lacked identifiable RBP sequences, suggesting an absence of canonical domains that can be detected using standard prediction tools.

**Discussion:**

These findings refine the classification of *Listeria* phages, significantly advancing our understanding of their taxonomy, genomic diversity, and global distribution.

## 1 Introduction

*Listeria monocytogenes* (Lm) is a foodborne pathogen that causes listeriosis, a rare but severe illness that primarily affects vulnerable populations, including children, the elderly, pregnant women, and immunocompromised individuals (Koopmans et al., 2023; Ravindhiran et al., 2023; Disson et al., 2025; Wong et al., 2024; Leclercq et al., 2024; Villa et al., 2025). The remarkable adaptability of Lm to a wide range of environmental conditions, including temperature, salt concentration, pH, and osmotic stress, poses significant challenges for food safety management (Disson et al., 2021; Tuytschaever et al., 2023; Manyi-Loh and Lues, 2025; Viazis et al., 2025; Grigore-Gurgu et al., 2024; Quereda et al., 2021; Li et al., 2025; Tsitsos et al., 2025; Sun et al., 2025; Zhang et al., 2025). *Listeria* phages are ubiquitous viruses that infect and lyse Lm with high specificity. They offer an alternative to traditional antibiotics without affecting the host's normal microbiota (Lavilla et al., 2023; Lasagabaster et al., 2020; Zbikowska et al., 2020; Abbas et al., 2022; Strathdee et al., 2023; Li et al., 2022). Phage biocontrol is increasingly recognized as a promising method of inhibiting Lm colonization and reducing the incidence of listeriosis (Li et al., 2022; Bumunang et al., 2023). Notably, the US FDA has approved two commercial products (Listex™ P100 and ListShield™) for *Listeria* biocontrol (Lavilla et al., 2023). Numerous studies have evaluated the efficacy of *Listeria*-specific phages in controlling Lm, with variable results (Liu et al., 2025; Vongkamjan et al., 2012).

It is evident that the majority of known phages are tailed and possess double-stranded DNA (dsDNA) genomes (Lavilla et al., 2023; Dion et al., 2020; Ackermann, 2007). Typically, phages are classified based on their life cycles into two distinct categories: lytic (virulent) phages and lysogenic (temperate) phages. Virulent phages follow a lytic cycle, which includes five critical stages: adsorption to the bacterial surface mediated by receptor-binding proteins (RBPs) that specifically recognize host cell surface receptors; injection of the phage genome into the host cytoplasm; biosynthesis of viral components, including the major capsid protein (MCP), which is essential for the assembly of the phage head; assembly and maturation of complete virions; and ultimately, host cell lysis. The final step is driven by phage-encoded endolysins, which enzymatically degrade the peptidoglycan layer of the bacterial cell wall, resulting in the release of progeny phages (Costa et al., 2025). Conversely, temperate phages may enter a lysogenic cycle, in which their genome integrates into the host chromosome and replicates along with it, facilitating stable maintenance and potential horizontal transmission (Dion et al., 2020; Ramos-Barbero et al., 2023).

To date, more than 500 *Listeria* phages have been identified (Lasagabaster et al., 2020). However, only a few virulent phages with biocontrol potential have been characterized in detail (Liu et al., 2025; Carlton et al., 2005; Hagens and Loessner, 2010; Schmuki et al., 2012). It is evident from the preceding studies that the genomic diversity and evolutionary relationships of *Listeria* phage have been explored, albeit with limited sample sizes (Dorscht et al., 2009; Denes et al., 2014). Since then a substantial number of new *Listeria* phage isolates have been reported (Song et al., 2019; Hudson et al., 2019; Erickson et al., 2021). The pronounced genetic mosaicism of phage genomes further complicates the reconstruction of their evolutionary relationships, underscoring the importance of structural and functional proteins in phage taxonomy and evolution (Dion et al., 2020; Denes et al., 2014; Pedulla et al., 2003). As demonstrated in recent phylogenomic analyses of *Listeria* phage (Liu et al., 2025; Schmuki et al., 2012), previous studies have primarily relied on the phylogenetic placement of newly isolated phages using conserved proteins with high sequence similarity. However, no systematic investigation has yet addressed the diversity and classification of these proteins. A comprehensive analysis of all publicly available *Listeria* phage genomes, coupled with comparative assessment of major capsid proteins (MCPs), receptor-binding proteins (RBPs), and endolysins, is therefore essential to refine our understanding of their genomic diversity and evolutionary dynamics across ecological and geographic contexts.

The aim of this study was to refine the systematic classification of *Listeria* phage by integrating publicly available genomic data with that from 16 newly sequenced phages. Furthermore, the diversity and distribution of three key structural and functional proteins (MCP, RBP, and endolysin) across different phage clusters were investigated. This investigation was undertaken to enhance our understanding taxonomy, genomic diversity, and global distribution of *Listeria* phage in ecosystems.

## 2 Material and methods

### 2.1 Genome collection and processing of *Listeria* phage

The total of 97 *Listeria* phage genomes was included, of which 81 were obtained from NCBI and 16 were newly sequenced in this study. The distribution of the phages isolated from 14 countries encompasses North America, South America, Europe and Asia.

#### 2.1.1 NCBI genome data collection

Initially, 166 sequences were retrieved from the National Center for Biotechnology Information (NCBI) database using the search query: ((“*Listeria monocytogenes* bacteriophage” [Title] OR “*Listeria* phage” [Title]) OR (“*Listeria monocytogenes* bacteriophage” [Organism] OR “*Listeria* phage” [Organism]) OR (“*Listeria monocytogenes* bacteriophage” [Source] OR “*Listeria* phage” [Source])) AND “viruses” [Filter] AND (30000 [SLEN]: 999999999[SLEN]) AND “complete genome” [Title], as of April 2025. Concurrently, associated metadata, encompassing sample source and geographic origin, were also retrieved from NCBI for each sequence. These data were then corroborated against the primary literature for the respective phage. Following a thorough manual verification process to ensure the integrity and relevance of the sequences, 115 sequences were confirmed as valid *Listeria* phage genomes. Whole-genome alignments and taxonomic identifier (taxid) analyses were conducted to remove redundant sequences, yielding 81 unique genomes.

#### 2.1.2 DNA extraction and genome sequencing of 16 newly isolated phages

16 representative phages were selected from the 317 *Listeria* phage in our previous study (Chen et al., 2025), based on their lytic profiles and ecological origins. To maximize diversity, phages were chosen to reflect variation in host range across 35 Lm strains, as well as diversity in sample sources and geographic origins. The 317 phages were classified into Broad, Medium, and Narrow Host Range Phages (BHRP, MHRP, and NHRP), from which 8, 4, and 4 phages were selected, respectively. This ensured that the selected phages captured the major phenotypic and genotypic diversity of the full collection (Chen et al., 2025). Genomic DNA of the phage was extracted using the Phage Genome Extraction Kit (ABigen Corporation, Beijing, China), following the manufacturer's instructions. Whole-genome sequencing was performed on the Illumina NovaSeq PE150 platform (MagiGene, Guangzhou, China). The procedures pertaining to the sequencing and genome assembly were conducted in accordance with protocols that had previously been described in detail (Liu et al., 2025).

### 2.2 Confirmation of phage genome characteristics

The lifestyles (virulent or temperate) of the 97 phages were initially predicted using PhaTYP (a tool of PhaBOX) and subsequently refined through a review of published literature (Shang et al., 2023). The calculation of genome lengths and GC contents was conducted utilizing the Seqkit software, which was operated within the Linux environment. For the 81 phages sourced from NCBI, the ICTV taxonomic classifications were obtained directly based on NCBI taxids. For the remaining 16 newly sequenced phages, ICTV classifications were predicted by PhaGCN (a tool of PhaBOX). ORF and tRNA counts were extracted from NCBI “gene features” files when available. Genomes devoid of annotations, in addition to all newly isolated phages, were subjected to uniform annotation using Prokka. ORF counts were subsequently summarized. tRNAs were detected using tRNAscan-SE. The prediction of auxiliary metabolic genes (AMGs) was conducted utilizing the DRAM-v software (Shaffer et al., 2020). The identification of antibiotic resistance genes was conducted through the utilization of the RGI approach, which was employed to predict proteins against the CARD database. The identification of virulence factors was facilitated by the DIAMOND blastp method, which was used to query the VFDB database. All analysis were conducted utilizing the default parameters.

### 2.3 Identification of representative proteins and conserved domains

Genes encoding major capsid proteins (MCPs) and endolysins were identified from existing genome annotations and validated using BLASTp (coverage >60%; identity >60%). The identification of receptor-binding proteins (RBPs) was initiated through a comparative analysis with previously reported *Listeria* phage RBPs. This comparison was then confirmed via BLASTp (coverage >60%; identity >60%), and the results were further validated using HHpred (E-value <1e-5). For 23 phages, of which no BLASTp match to known RBPs was found, predictions were based exclusively on HHpred: 4 phages yielded 3 RBP candidates each, 12 phages yielded 2 candidates each, and 7 phages yielded 0 candidates. In instances where HHpred returned multiple candidates for a given phage, we retained the RBP showing the greatest number of relevant functional domain hits, the longest aligned region, and the lowest E-value was retained for downstream RBP diversity analyses. Pairwise amino acid identity among selected proteins was calculated using EMBOSS software in a Linux environment. The annotation of conserved domains within these proteins was achieved through querying the NCBI Conserved Domain Database (CDD) (https://www.ncbi.nlm.nih.gov/cdd) and the InterPro database (https://www.ebi.ac.uk/interpro/). In instances where domain annotations from both databases overlapped by more than 70%, annotations from CDD were prioritized.

### 2.4 Construction of proteomic and phylogenetic trees

A proteomic tree based on genome-wide sequence similarity was generated using the ViPTree server (https://www.genome.jp/viptree/). Phylogenetic analyses for MCP, RBP, and endolysin sequences were conducted by aligning nucleotide sequences with MAFFT v7.407, followed by trimming ambiguously aligned regions using TrimAl v1.2. Phylogenetic trees were subsequently constructed using IQ-TREE v1.6.9, employing the optimal substitution models selected automatically by ModelFinder with the “-m MFP” option. The processes of visualization and annotation of trees were conducted utilizing the software programs FigTree and ChiPlot.

### 2.5 Phenotypic characterization of 16 newly isolated phages

#### 2.5.1 Storage stability testing and phage titer determination

The phage stocks were stored at a temperature of 4 °C. The phage titer was monitored at three-month intervals over a 12-month period using the standard double-layer agar assay. In summary, 100 μL of host bacterial culture was mixed with 3-5 mL of molten top agar (0.7% agar, maintained at approximately 45 °C) and poured onto agar plates, which were then left to solidify at room temperature. Phage suspensions were subjected to ten serial dilutions in Brain Heart Infusion (BHI) broth. For each dilution step, 20 μL of phage solution was mixed into 180 μL of BHI broth. Subsequently, 10 μL of each dilution was spotted onto the solidified soft agar surface. The plates were then subjected to an overnight incubation at 30 °C. Following the incubation period, the number of plaques present on each plate was enumerated, with the plates containing 10-100 plaques being counted. The phage titer was calculated using the following formula: The phage titer (PFU/mL) = Number of plaques × Dilution × 100.

#### 2.5.2 Host range determination

The host ranges of 16 newly isolated phages were evaluated against 35 *Listeria monocytogenes* strains representing nine serotypes using the standard double-layer agar method, following protocols previously described (Liu et al., 2025). Each phage suspension was individually spotted onto a bacterial lawn, and the resulting plaques were assessed after overnight incubation at 30 °C. The lysis profiles were categorized as follows: clear plaque formation, turbid plaque formation, or no plaque formation.

## 3 Results

### 3.1 Basic features of *Listeria* phage

#### 3.1.1 Sample sources and geographic distribution

Of the 97 *Listeria* phage analyzed, 63 were recovered by direct isolation from samples, 9 by induction of lysogenic strains, 2 were derived from previously characterized phages, and 23 from unknown sources (Supplementary Table S1). Among the directly isolated phages, sampling sites were predominantly food-associated environments, including meat-processing plant drains, conveyor belts, floor drains, wastewater and slaughterhouse sewage, retail-market swabs, fowl droppings, cattle feces, and farm silage, as well as food products, including chicken meat and skin, cheese, seafood, frozen foods, and wild mushrooms/mushroom compost. Additional isolates were obtained from natural environments (sand and soil) and from clinical samples. The geographical origins of the isolates are as follows: North America (Canada, *n* = 9; United States, *n* = 20), South America (Argentina, *n* = 2), Europe (Ireland, *n* = 1; Germany, *n* = 4; France, *n* = 2; Switzerland, *n* = 2; Spain, *n* = 1; Hungary, *n* = 1; United Kingdom, *n* = 1), and Asia (South Korea, *n* = 2; Japan, *n* = 3; Thailand, *n* = 4; China, *n* = 19), with 26 phages of unknown geographic origin (Supplementary Table S1).

#### 3.1.2 Genome characteristics and taxonomic classification

The analysis of 97 *Listeria* phage revealed that they possess linear double-stranded DNA genomes ranging in size from 32,895 to 223,580 base pairs. The distribution of genome sizes of the identified phages revealed 45 phages (46.39%) with large genomes (131–224 kb), 11 (11.34%) with medium genomes (64–87 kb), and 41 (42.27%) with small genomes (35–48 kb). Predicted open reading frame (ORF) counts ranged from 53 to 229 per genome. The range of GC content was from 25.92% to 45.51% (mean ± SD, 36.18% ± 1.61%). Of the 97 phages analyzed, 58 were predicted to be virulent and 39 temperate. According to the most recent ICTV classification, 61 phages were assigned to the genus level: 43 to the genus *Pecentumvirus* (including 13 unclassified *Pecentumvirus*; 174 -206 open reading frames [ORFs]; GC content 35.96% ± 0.08%; all encoding 10–17 transfer RNA [tRNAs]), 10 to the genus *Homburgvirus* (108-119 ORFs; GC content 36.39% ± 0.08%; no tRNAs), 4 to the genus *Slepowronvirus* (including 2 unclassified *Slepowronvirus*; 54–70 ORFs; GC content 35.09% ± 0.31%; no tRNAs), 2 to the genus *Psavirus* (61–69 ORFs; GC content 35.47% ± 1.05%; no tRNAs), and 1 each to the genus *Jelitavirus* and *Thornevirus* (65 and 217 ORFs, respectively; both lacking tRNAs). One additional phage was placed in the subfamily *Ounavirinae* but remained unclassified at the genus level (120 ORFs; 11 tRNAs). The remaining 35 phages were unclassified (unclassified bacterial viruses and unclassified *Caudoviricetes*), with 53–229 ORFs and GC content 36.60% ± 2.61%. It is noteworthy that LPJP1 possessed the most substantial genome (223,580 bp), encompassing 229 ORFs and 4 predicted tRNAs; the remaining members of this group were devoid of tRNAs (Supplementary Table S1).

Across these 97 phages, 62 (63.9%) were predicted to harbor auxiliary metabolic genes (AMGs). A total of eight AMGs were identified, including *cobS, dut, nrdA, nrdB, DNMT1, UGDH, thyA*, and *nrdD*. No phage carried annotated antibiotic resistance or virulence genes with confirmed functions, although putative homologs to such factors were detected.

#### 3.1.3 Phenotypic traits of 16 newly isolated phages

A plethora of phenotypic reports for *Listeria* phage retrieved from NCBI are available, encompassing transmission electron microscopy (TEM) morphology and host-range profiles. However, cross-study comparability is constrained by heterogeneity in the bacterial test panels employed. The TEM data for 16 of the aforementioned phages have been published (Chen et al., 2025). In order to further characterize these isolates, an assessment was made of the storage stability and host range of the 16 phages.

##### 3.1.3.1 Storage stability

The long-term storage stability of the 16 new isolated phages was evaluated over a 12-month period at 4 °C. Initial titers ranged from 1.40 × 10^9^ to 1.30 × 10^11^ PFU/mL. A general decline in titers was observed after three months, though levels remained relatively high. A gradual reduction was observed at 6 and 9 months; however, the titer levels remained within the acceptable limits. By the 12th month, the majority of phage titers were maintained within the range of 10^7^ to 10^9^PFU/mL, indicating good stability under refrigerated conditions (Table 1) and supporting their potential for long-term application.

**Table 1:** Long-term stability at 4 °C of 16 newly isolated phages.

**Name**	**0 month**	**3 month**	**6 month**	**9 month**	**12 month**
Phage17	1.35 × 10^10^	3.40 × 10^9^	3.90 × 10^8^	9.50 × 10^6^	3.90 × 10^5^
Phage19	1.50 × 10^10^	1.55 × 10^9^	4.45 × 10^8^	1.35 × 10^8^	1.50 × 10^7^
Phage33	1.40 × 10^9^	4.00 × 10^8^	2.45 × 10^8^	4.45 × 10^6^	3.00 × 10^6^
Phage39	1.50 × 10^10^	1.25 × 10^10^	6.00 × 10^9^	3.50 × 10^9^	3.00 × 10^9^
Phage62	1.10 × 10^10^	7.00 × 10^9^	1.30 × 10^9^	1.10 × 10^9^	1.30 × 10^8^
Phage102	4.00 × 10^10^	4.00 × 10^10^	1.50 × 10^10^	1.50 × 10^9^	8.00 × 10^8^
Phage130	1.25 × 10^11^	3.00 × 10^10^	6.50 × 10^9^	1.15 × 10^9^	3.50 × 10^8^
Phage144	9.00 × 10^10^	1.10 × 10^10^	6.00 × 10^9^	3.50 × 10^9^	1.15 × 10^9^
Phage189	6.00 × 10^10^	4.75 × 10^10^	8.50 × 10^9^	8.50 × 10^8^	5.00 × 10^6^
Phage201	4.30 × 10^9^	6.50 × 10^8^	1.30 × 10^8^	1.10 × 10^8^	6.50 × 10^7^
Phage208	1.30 × 10^11^	2.50 × 10^10^	5.00 × 10^9^	8.50 × 10^6^	6.00 × 10^6^
Phage211	2.15 × 10^10^	1.00 × 10^10^	8.50 × 10^9^	5.00 × 10^8^	1.05 × 10^8^
Phage222	1.50 × 10^10^	2.50 × 10^9^	1.45 × 10^9^	5.00 × 10^8^	3.00 × 10^7^
Phage225	6.50 × 10^10^	4.50 × 10^9^	1.60 × 10^9^	4.35 × 10^8^	2.15 × 10^7^
Phage251	1.65 × 10^10^	3.00 × 10^9^	6.00 × 10^8^	3.50 × 10^8^	1.35 × 10^8^
Phage263	2.60 × 10^10^	9.50 × 10^9^	6.00 × 10^9^	1.15 × 10^9^	3.35 × 10^8^

##### 3.1.3.2 Host-range test

**Figure 1:**
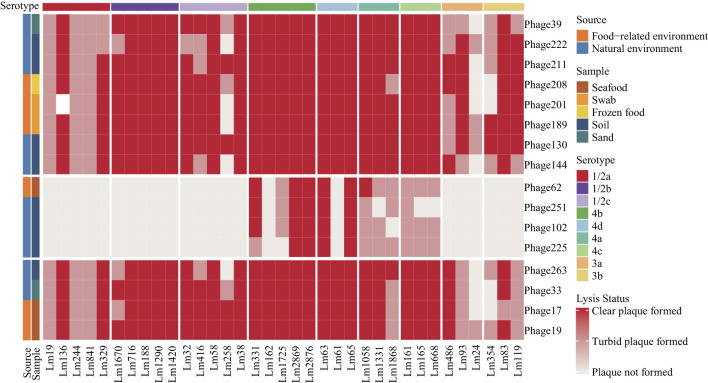
Host range of 16 newly isolated phages against 35 *Listeria monocytogenes* strains determined by the standard double-layer agar method. Left-side labels indicate the sources and sample types of the phages. Each column represents a host strain, annotated with its serotype.

As outlined in the previous study (Chen et al., 2025), the province of origin and sample sources of 16 newly isolated phages have been delineated. The lytic spectrum of the 16 phages was determined using the standard double-layer agar method (Figure 1), with 35 Lm strains were used. The lytic activity of the phages was found to be most effective against serotype 4 strains. However, only a few phages were capable of lysing strains Lm24 (serotype 3a), Lm258 (serotype 1/2c), and Lm354 (serotype 3b), while none were able to efficiently lyse the serotype 1/2a strains Lm19, Lm244, and Lm841. It is noteworthy that the four narrow-host-range phages (Phage62, Phage102, Phage225, and Phage251) seem to demonstrate specificity toward serotype 4 strains.

### 3.2 Whole-genome-based phylogenetic analysis reveals nine distinct phage clusters

**Figure 2:**
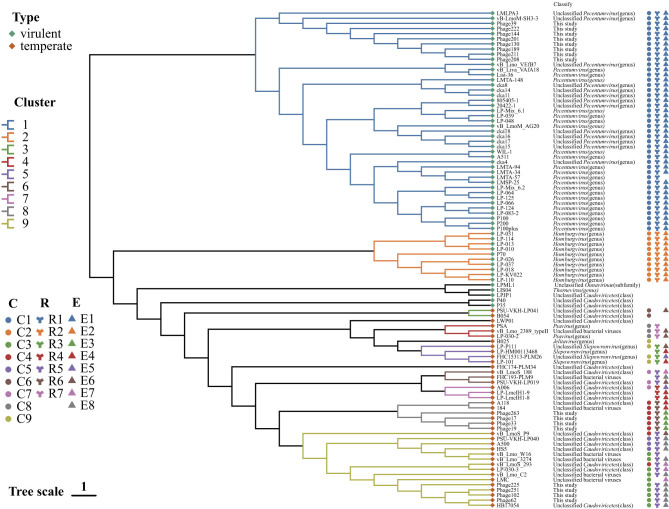
Phylogenetic relationships of *Listeria* phage based on whole-genome analysis. A viral proteomic tree was constructed using Viptree based on the whole genomes of 97 *Listeria* phages (81 retrieved from NCBI and 16 newly isolated in this study). Using a distance threshold of 0.27, the phages were grouped into nine distinct clusters. Taxonomic assignments follow the official ICTV classification and are indicated after each phage name. Annotations on the right indicate the classification of the corresponding MCP, RBP, and endolysin proteins (for detailed descriptions, see Figures 4–6).

**Figure 3:**
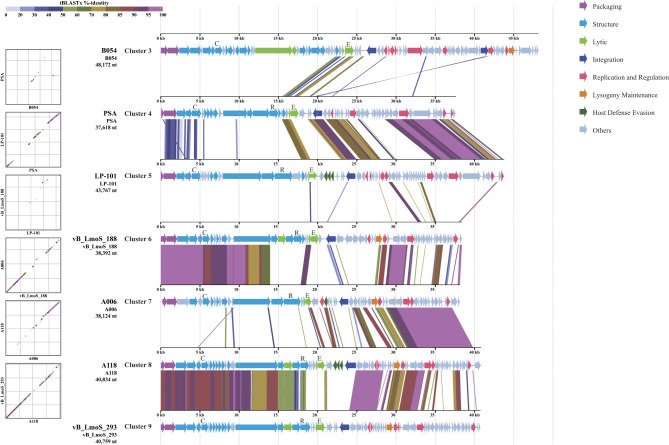
Genomic comparison of seven representative phages from each temperate phage cluster. CDS alignments of representative genomes from seven temperate phage clusters (Clusters 3–9) are shown. Arrows indicate open reading frames (ORFs), with colors representing distinct gene categories classified into eight functional groups: Packaging, Structure, Lytic, Integration, Replication and Regulation, Lysogeny Maintenance, Host Defense Evasion, and Others. Representative genes (MCP, RBP, and endolysin) are briefly annotated as C, R, and E, respectively. Colored bands connecting the genetic maps illustrate sequence identity percentages between genomes, as indicated by the color gradient in the legend at the top left of the figure.

As demonstrated in Figure 2, the application of proteome tree to the 97 *Listeria* phage resulted in the grouping of these into nine clusters. In addition to these, eight genomes were identified as unclustered singletons. Cluster 1 (*n* = 43) and Cluster 2 (*n* = 10) comprised virulent phages; five additional virulent phages were singletons. Conversely, Cluster 3-Cluster 9 and three singletons were classified as temperate phages exhibiting greater diversity. Phage B054, PSA, LP-101, vB_LmoS_188, A006, A118 and vB_LmoS_293 was selected as representative temperate phages of Cluster 3, 4, 5, 6, 7, 8, 9 for comparative analysis (Figure 3), whole-genome similarity across these seven clusters was found to be limited, consistent with pronounced divergence among temperate phages (Dion et al., 2020). Nevertheless, conservation was evident in the structural gene modules, it is relatively similar between Cluster 6 and Cluster 7, Cluster 8 and Cluster 9; replication/regulatory (and some unclassified) modules were notably similar between Cluster 4 and Cluster 5, Cluster 7 and Cluster 8, and Cluster 8 and Cluster 9; and integration-related modules showed marked similarity between Cluster 4 and Cluster 5 (Figure 3).

All *Pecentumvirus* phages were found to fall within Cluster 1 (n = 43), including eight newly isolated broad-host-range virulent phages (Phage39, Phage130, Phage144, Phage189, Phage201, Phage208, Phage211, and Phage222). These eight phages exhibited a high degree of nucleotide identity to *Listeria* phage vB-LmoM-SH3-3 (ANI 99.1489-99.1551%; Supplementary Figure S1A). The isolation sources that were made available for 33/43 Cluster 1 phages and included sources of sewage, wastewater, meat-processing effluents and drains, poultry-farm effluents, sand, soil, swabs, frozen food, silage, and food-processing environments (floors, conveyor belts, drains). The geographic distribution include North America (*n* = 20), South America (*n* = 2), Europe (*n* = 2), and Asia (*n* = 10). The TEM morphology of 16 Cluster 1 phages was reported, all of which were found to be *Myoviridae*-like (Chen et al., 2025). All *Homburgvirus* phages formed Cluster 2 (*n* = 10), were predominantly silage sources (9/10). The geographical distribution of the isolates included North America (*n* = 8), Europe (n = 1), and Asia (*n* = 1). Based on the TEM images, eight Cluster 2 phages were found to be *Siphoviridae*-like, typically displaying elongated capsids (Song et al., 2019; Hudson et al., 2019). Among the five virulent singletons, an unclassified *Ounavirinae* LPML1 (Asia) and a *Thornevirus* phage (Asia, paddy soil) were identified as *Siphoviridae*-like by TEM. Three other viruses were classified within the unclassified *Caudoviricetes* category: LPJP1, the largest *Listeria* phage reported to date, was isolated from North American silage and was found to be *Myoviridae*-like; P40 (Europe) and P35 (silage) were *Siphoviridae*-like (Dorscht et al., 2009; Erickson et al., 2021).

The majority of temperate phages were found to be taxonomically unassigned, categorized as either unclassified bacterial viruses or unclassified *Caudoviricetes*. The following genera were represented: Cluster 4 (*n* = 3) comprised two *Psavirus* phages and one unclassified phage. Phage PSA was induced from a lysogenic strain in Europe and exhibited *Siphoviridae*-like morphology (Denes et al., 2014); Phage LP-030-2, isolated from farm silage in North America, was also *Siphoviridae*-like (Denes et al., 2014); and the unclassified phage vB_Lmo_2389_typeII originated from a European sewage treatment plant. Four *Slepowronvirus* phages were grouped in Cluster 5; two were isolated in Asia and North America (cattle feces and farm silage), and one has *Siphoviridae*-like morphology by TEM (Denes et al., 2014). The *Jelitavirus* phage B025 was a singleton, having been induced from an *L. innocua* lysogen and displayed *Siphoviridae*-like morphology (Dorscht et al., 2009). The remaining temperate clusters lack an established genus-level classification under the ICTV taxonomy. Cluster 3 comprised two induction-derived phages, one of which was Asian origin. Cluster 6 comprised one induction-derived phage from Asia and two food-related isolates (wild mushroom and chopped chicken meat), originating from Asia and an unknown location, respectively. Cluster 7 comprised one inducible phage displaying *Siphoviridae*-like morphology, in addition to two phages derived from clinical isolates (Dorscht et al., 2009). Cluster 8 contained *Listeria* phage A118 (lysogen-induced) and 184 (isolated in Europe), together with four newly isolated broad-spectrum temperate phages (Phage263, Phage17, Phage33, and Phage19), each isolated from various environments in Asia (soil, sand, or seafood). These four phages occupied distinct phylogenetic branches, indicating substantial evolutionary divergence. Among them, phage 184 exhibited the highest genomic similarity to Phage17, Phage33, and Phage19 (ANI = 93.72-95.67%), whereas Phage263 was the most divergent member of the cluster (ANI = 93.72%) (Supplementary Figure S1B). Cluster 9 (*n* = 15), the largest temperate phage cluster, included two lysogen-induced phages and four newly isolated serotype 4-specific narrow-spectrum phages (Phage62, Phage102, Phage225, Phage251). These four phages exhibited extremely high genomic similarity to *Listeria* phage HB17054 (ANI 99.8689-99.9739), indicating a close evolutionary relationship (Supplementary Figure S1C). The sources of Cluster 9 phages encompassed seafood, cheese, mushroom compost, silage, avian droppings, food-processing environments, soil, and sewage treatment plants, with isolates from North America (*n* = 1), Europe (*n* = 4), and Asia (*n* = 6). Two taxonomically unassigned temperate singletons, one was isolated from chicken skin (Asia) and the other one from Europe.

The host-range of 48 phages (32 virulent and 16 temperate) was demonstrated (Supplementary Table S1). The occurrence of broad-host-range phages was identified in Cluster 1, Cluster 2, Cluster 7, and Cluster 8. The host range of 25/43 phages in Cluster 1 was reported; the eight newly isolated virulent members exhibited a broad host range. In general, Cluster 1 phages frequently lysed *L. monocytogenes* serotypes 1/2 and 4, with some also infecting *L. innocua* and *L. welshimeri*; vB_Lino_VEfB7 was an exception, infecting only *L. innocua* (Blanco Fernandez et al., 2021). Host-range of 3/10 Cluster 2 phages was heterogeneous; two were broad (lysing serotypes 1/2, 4, 5, and 6), whereas one was narrow (limited to serotypes 6a and 6b). Among virulent singletons phages with host-range data, LIS04 infected *L. innocua* (Park and Chang, 2025); LPJP1 lysed *L. grayi* (Erickson et al., 2021); P40 exhibited a broad host range (serotypes 1/2, 4, 5, and 6); and P35 targeted serotype 1/2 (Dorscht et al., 2009). For the temperate phage clusters, B054 (Cluster 3) exhibited targeting of serotypes 5 and 6; PSA (Cluster 4) demonstrated lysis of serotype 4b; vB_LmoS_188 (Cluster 6) exhibited targeting of serotypes 4b and 4e; and A006 (Cluster 7) exhibited targeting of serotype 1/2. In Cluster 8 phages, A118 lysed serotype 1/2 strains, and the four newly isolated temperate phages exhibited a broad spectrum of activity (Loessner, 1991). In Cluster 9, phage A500, vB_LmoS_293, and four newly isolated phages exhibited narrow host ranges that were restricted to serotype 4 *L. monocytogenes*. The singleton phage B025 lysed serotypes 5 and 6 (Loessner, 1991).

Furthermore, the composition of AMG varied considerably across clusters. Cluster 1 encoded four AMGs (*cobS, dut, nrdA, nrdB*) associated with purine, pyrimidine, and porphyrin metabolism. Cluster 9 exclusively encoded *DNMT1*, which is linked to cysteine and methionine metabolism, while Cluster 2 carried only *cobS*, which is involved in porphyrin metabolism. Among singleton virulent phages, the phage LIS04 was found to encode *nrdA, nrdB*, and *UGDH*, which contributing to the biosynthesis of purine, pyrimidine, carbohydrate, and glycan biosynthesis pathways. Whereas, the phage LPML1 encoded *nrdB, thyA*, and *nrdD*, which were found to be involved in the metabolism of nucleotide, cofactor-vitamin, purine, and pyrimidine.

### 3.3 Phylogenetic typing and domain analysis of major capsid protein, receptor-binding protein and endolysin

**Figure 4:**
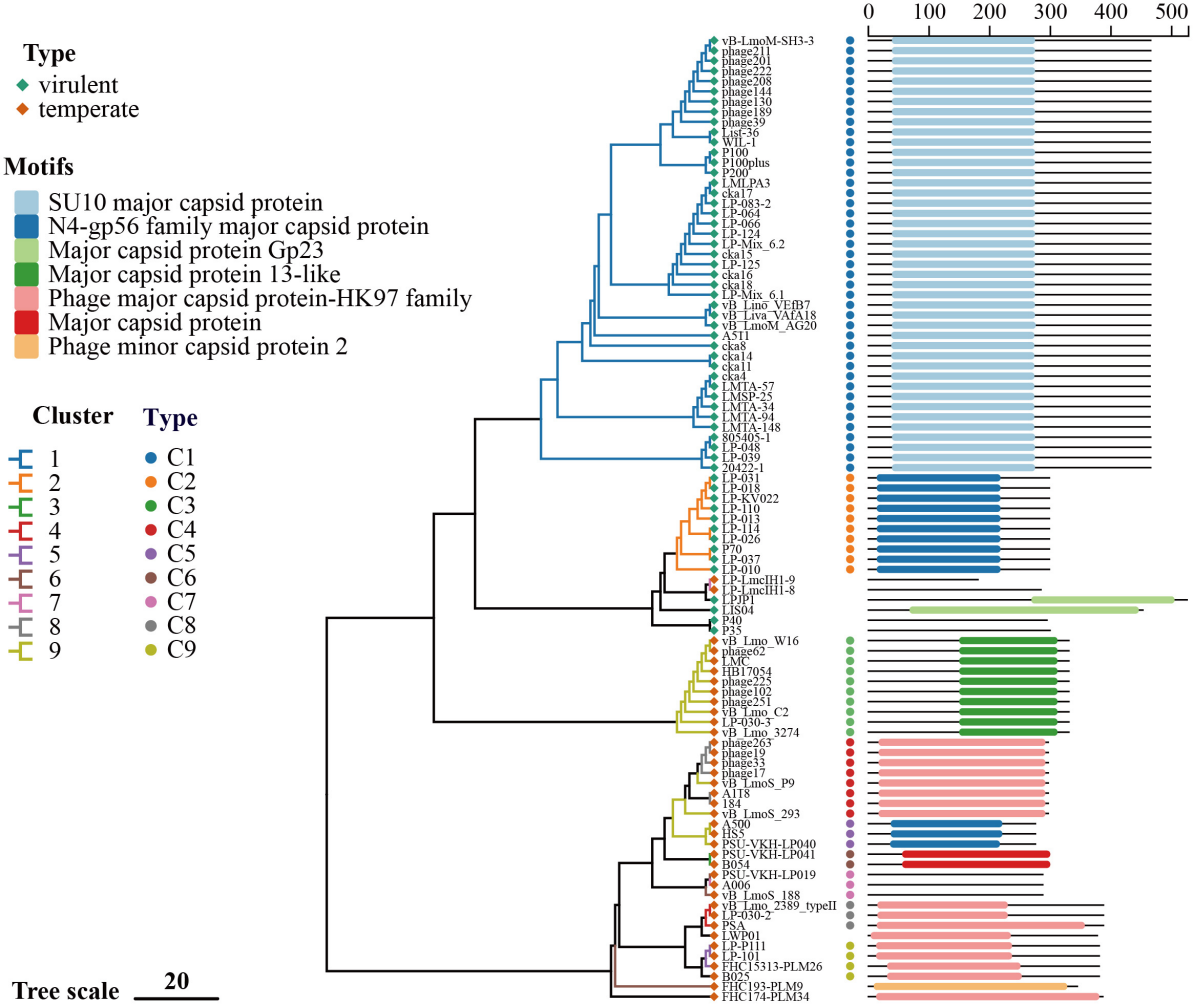
Phylogenetic analysis and conserved domain annotation of MCP from *Listeria* phage. A phylogenetic tree was constructed using IQ-TREE based on nucleotide sequences of the MCP gene from 95 *Listeria* phages. The conserved domain classifications from the CDD are annotated alongside each phage. Scale bar represents nucleotide substitutions per site.

**Figure 5:**
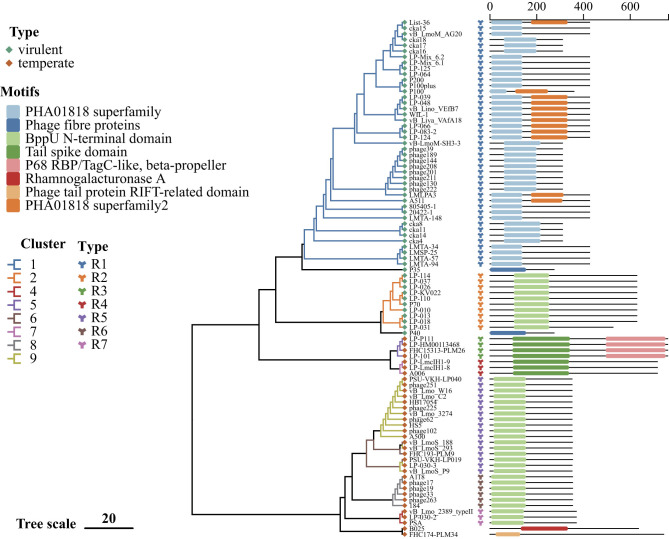
Phylogenetic analysis and conserved domain annotation of RBP from *Listeria* phage. A phylogenetic tree was constructed using IQ-TREE based on nucleotide sequences of the RBP gene from 90 *Listeria* phages. The conserved domain classifications from the CDD are annotated alongside each phage. Scale bar represents nucleotide substitutions per site.

**Figure 6:**
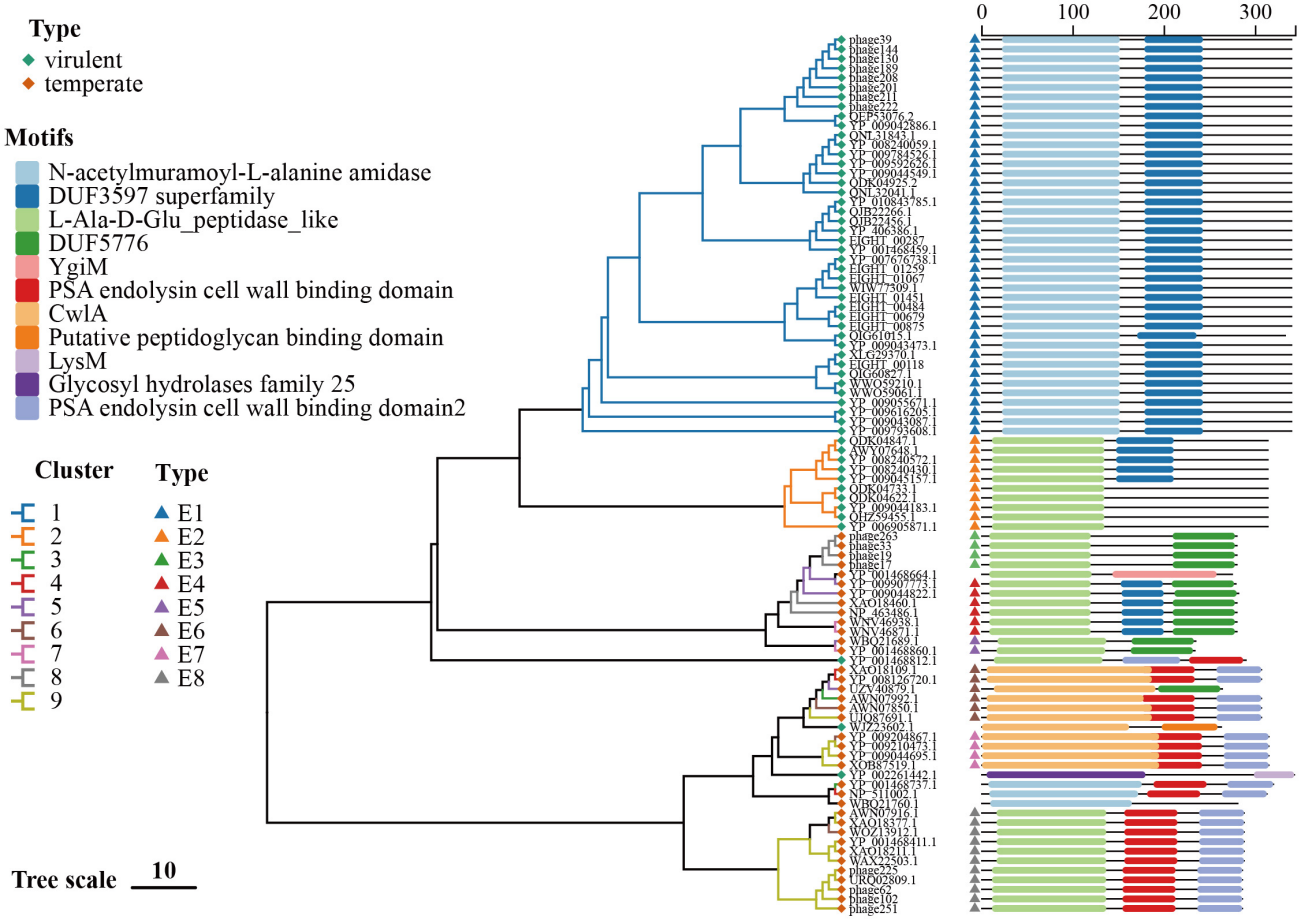
Phylogenetic analysis and conserved domain annotation of endolysin from *Listeria* phage. A phylogenetic tree was constructed using IQ-TREE based on nucleotide sequences of the endolysin gene from 92 *Listeria* phages. The conserved domain classifications from the CDD are annotated alongside each phage. Scale bar represents nucleotide substitutions per site.

In order to investigate the diversity and evolutionary relationships among the identified *Listeria* phage, three representative genes coding for major capsid protein (MCP), receptor-binding protein (RBP), and endolysin were selected for phylogenetic analysis and are denoted as C, R, and E in the figures. The corresponding nucleotide sequences were extracted from the 97 phage genomes undertaken, and the phylogenetic trees were constructed separately for each gene (Figures 4–6).

#### 3.3.1 Major capsid protein (MCP)

MCP sequences were identified in 95 of the 97 genomes and classified into nine types, with nine sequences designated as singletons (Figure 4, Table 2). Types C1 and C2 were found to be associated with virulent phages, whereas Types C3–C9 were found in temperate phages, which exhibited greater MCP diversity. Type C1 was the most prevalent, concentrated in Cluster 1 and accounting for 44.33% of all known *Listeria* phage MCPs. Type C2 occurred in Cluster 2. The additional distributions were observed: Type C3 and Type C5 in Cluster 9; Type C4 in Clusters 8 and 9; Type C6 in Cluster 3; Type C7 in Clusters 6 and 7; Type C8 in Cluster 4; and Type C9 in Cluster 5. MCPs in Cluster 9 were found to be heterogeneous, encompassing Types C3, C4, and C5. The majority of MCPs exhibited a single domain annotation, although a subset exhibited undetectable hits. The domains detected included SU10 major capsid protein; N4-gp56 family major capsid protein; major capsid protein Gp23; major capsid protein 13-like; phage major capsid protein-HK97 family; major capsid protein; and phage minor capsid protein 2. These domains have been shown to be associated with the canonical HK97-fold capsid architecture, as has been previously demonstrated in other studies (Duda and Teschke, 2019; Šiborová et al., 2022; Rao et al., 2023; Podgorski et al., 2023).

**Table 2:** Classification of MCP based on phylogenetic analysis and amino acid identity.

**Type/Phage**	**Phage cluster**	**Domain**	***N***	**Identity (%)**
C1	1	SU10 major capsid protein	43	98.90–100
C2	2	N4-gp56 family major capsid protein	10	99.70–100
C3	9	Major capsid protein 13-like	10	100
C4	8, 9	Phage major capsid protein-HK97 family	8	91.60–100
C5	9	N4-gp56 family major capsid protein	3	89.20–100
C6	3	Major capsid protein	2	96.7
C7	6, 7	–	3	86.6–100
C8	4	Phage major capsid protein-HK97 family	3	90–100
C9	5, S	Phage major capsid protein-HK97 family	4	91.1–100
LP-LmcIH1-9, LP-LmcIH1-8	7	–	2	63.80
LPJP1	S	Major capsid protein Gp23	1	–
LIS04	S	Major capsid protein Gp23	1	–
P40, P35	S	–	2	59.70
LWP01	S	Phage major capsid protein-HK97 family	1	–
FHC193-PLM9	6	Phage minor capsid protein 2	1	–
FHC174-PLM34	S	Phage major capsid protein-HK97 family	1	–

If an MCP could not be assigned to any defined type, the corresponding phage name is directly listed in the first column as an outlier. The “Phage Cluster” column indicates the whole-genome-based classification of the phage: numbers 1–9 represent Cluster1 to Cluster9, and “S” denotes singleton.

#### 3.3.2 Receptor-binding protein (RBP)

A total of 90 RBPs were identified and subsequently classified into seven distinct types, in addition to four singletons (Figure 5, Table 3). These groupings generally corresponded to genome clusters. Types R1, R2, R4, and R6, which have been linked to a broad host range, spanned Clusters 1, 2, 7, and 8 phages. Types R1 and R2 were found to be associated with virulent phages, whereas types R3-R7 were identified in temperate phages. Type R1, representing 47.78% of identified RBPs, was confined to Cluster 1 phage and included the well-characterized RBP from phage A511, known for its broad host range (lytic against serotypes 1/2, 4, 5, and 6 strains). Type R2 (Cluster 2 phage) currently lacks experimentally characterized RBPs, predictions were derived from HHpred, although the two corresponding phages have been reported as broad-host-range. Type R3, identified in Cluster 5 phages, also lacks characterization, with RBPs predicted via HHpred. Type R4, identified in Cluster 7 phages, encompasses the extensively studied RBP of A006, which targets serotype 1/2 strains. Type R5 (Cluster 6 and 9) comprises RBP from phages vB_LmoS_293 and vB_LmoS_188, both of which are specific to serotype 4. Type R6, as classified within Cluster 8 phages, encompasses A118, which has been documented as serotype 1/2-specific but has also been observed to include newly identified broad-host-range temperate phages (lytic to serotype 4). Type R7 is restricted to Cluster 4 phages (encompasses PSA), which has been demonstrated to infect serotype 4b strains. No known RBP was identified in Cluster 3 phages. Domain annotations for RBPs included the PHA01818 superfamily, BppU N-terminal domain, Tail spike domain, P68 RBP, Phage tail protein RIFT-related domain, and Rhamnogalacturonase A.

**Table 3:** Classification of RBP based on phylogenetic analysis and amino acid identity.

**Type/phage**	**Phage cluster**	**Domain 1**	**Domain 2**	***N***	**Representative phage**	**Identity (%)**
R1	1	PHA01818 superfamily	–	43	A511	60.5–100
R2	2	BppU N-terminal domain	–	10	–	76.6–100
R3	5	Tail spike domain	P68 RBP	4	–	93.1–98.7
R4	7	Tail spike domain	–	3	A006	97.1–100
R5	6, 9	BppU N-terminal domain	–	17	vB_LmoS_188, vB_LmoS_293	91.3–100
R6	8	BppU N-terminal domain	–	6	A118	100
R7	4	BppU N-terminal domain	–	3	PSA	96.0–100
P35	S	–	–	–	P35	–
P40	S	–	–	–	–	–
B025	S	Rhamnogalacturonase A	–	1	B025	–
FHC174-PLM34	S	Phage tail protein RIFT-related domain	–	1	–	–

If a RBP could not be assigned to any defined type, the corresponding phage name is directly listed in the first column as an outlier. The “Phage Cluster” column indicates the whole-genome-based classification of the phage: numbers 1–9 represent Cluster1 to Cluster9, and “S” denotes singleton. “Representative phage” refers to *Listeria* phage with well-characterized receptor-binding proteins (RBPs) that have been confirmed in previous studies.

#### 3.3.3 Endolysin

A total of 92 endolysins were identified, which were subsequently classified into eight distinct types and seven singletons (Figure 6, Table 4). Types E1 and E2 were found to be associated with virulent phages, while types E3-E8 were identified within temperate phages. The most prevalent lineage was type E1, concentrated in Cluster 1 and accounting for 44.30% of all known *Listeria* phage endolysins. Type E2 was observed in Cluster 2. In contrast, endolysins from temperate-phage clusters exhibited greater diversity: Cluster 3 (Type E6); Cluster 4 (Type E6); Cluster 5 (Types E4, E5, E6); Cluster 6 (Types E6, E7, E8); Cluster 7 (Types E4, E5); Cluster 8 (Types E3, E4); and Cluster 9 (Types E6, E7, E8). The majority of endolysins were found to carry two or three conserved domains, while a minority encoded only one. The domains identified in this study included the catalytic N-acetylmuramoyl-L-alanine amidase, L-Ala-D-Glu peptidase-like, GH25, and LysM domains, as well as the PSA endolysin CBD. The additional domains identified in this study included PG_binding_1, DUF3597, and DUF5776. PG_binding_1 is regarded as a general, albeit low-evidence, cannabidiol (CBD) candidate (Korndörfer et al., 2006, 2008; Romero et al., 2018). It has been established that other hits, such as CwlA and YgiM, were related to PG-binding; however, they lacked *Listeria* specificity.

**Table 4:** Classification of endolysin based on phylogenetic analysis and amino acid identity.

**Type/phage**	**Phage cluster**	**Domain 1**	**Domain 2**	**Domain 3**	***N***	**Identity (%)**
E1	1	N-acetylmuramoyl-L-alanine amidase	DUF3597 superfamily	–	42	95.9–100
E2	2	L-Ala-D-Glu_peptidase_like	DUF3597 superfamily (partially contained)	–	10	98.4–100
E3	8	L-Ala-D-Glu_peptidase_like	DUF5776	–	4	100
E4	5, 7, 8	L-Ala-D-Glu_peptidase_like	DUF3597 superfamily	DUF5776	6	83.7–100
E5	5, 7	L-Ala-D-Glu_peptidase_like	DUF5776	–	2	96.2
E6	3,4,5,6,9	CwlA	PSA endolysin cell wall binding domain / DUF5776	–	6	55.1–100
E7	6, 9	CwlA	PSA endolysin cell wall binding domain	–	4	94.6–98.1
E8	6, 9	L-Ala-D-Glu_peptidase_like	PSA endolysin cell wall binding domain	–	11	90.3–100
B025	S	L-Ala-D-Glu_peptidase_like	YgiM	–	1	–
P35	S	L-Ala-D-Glu_peptidase_like	PSA endolysin cell wall binding domain	–	1	–
LIS04	S	CwlA	Putative peptidoglycan binding domain	–	1	–
P40	S	Glycosyl hydrolases family 25	LysM	–	1	–
B054, PSA	3, 4	N-acetylmuramoyl-L-alanine amidase	PSA endolysin cell wall binding domain	–	2	46.4
FHC174-PLM34	S	N-acetylmuramoyl-L-alanine amidase	–	–	1	–

If an endolysin could not be assigned to any defined type, the corresponding phage name is directly listed in the first column as an outlier. The “Phage Cluster” column indicates the whole-genome-based classification of the phage: numbers 1–9 represent Cluster 1 to Cluster 9, and “S” denotes singleton.

## 4 Discussion

In light of the pervasive threat posed by *Listeria monocytogenes* to food safety and public health, there is an imperative for the isolation of novel phages and the comprehensive classification and evolutionary characterization of *Listeria* phage (Viazis et al., 2025; Li et al., 2025; Niu et al., 2024). Such efforts are crucial for the advancement of phage-based biocontrol strategies (Strathdee et al., 2023; Li et al., 2022; Peng et al., 2025). This study was the first to establish a classification system comprising nine distinct clusters based on the analysis of all *Listeria* phage complete genomes available in the NCBI database, representing isolates from 14 countries across four continents. Furthermore, the major capsid protein (MCP), receptor-binding protein (RBP), and endolysin were categorized into nine, seven, and eight types, respectively. The conserved domain profiles and distribution patterns of these three proteins were further characterized across the nine defined phage clusters.

This study provides a comprehensive summary of all available complete genomes of *Listeria* phage and classifies them into clusters based on whole-genome similarity. Despite the endeavors of preceding studies to cluster *Listeria* phage, their efforts were constrained by diminutive sample sizes and are now obsolete due to the accelerated identification of novel phages in recent years (Dorscht et al., 2009; Denes et al., 2014). 25 *Listeria* phage were categorized into five distinct orthoclusters in a previous study (Denes et al., 2014), the Orthocluster 1 and 5 corresponded precisely to Cluster 1 and Cluster 2 in our study. Therefore, the incorporation of a more extensive and varied collection of *Listeria* phage, permitted a more sophisticated classification of phages within Orthoclusters 2, 3, and 4. Phages previously grouped together despite considerable genomic divergence were reclassified into more genetically coherent clusters in this study, highlighting the genomic heterogeneity within these orthoclusters and offering a more comprehensive understanding of *Listeria* phage diversity and taxonomy. In the previous study, A006, A118, and A500 were grouped together, PSA was placed with B025, while P35 and P40 were classified as a separate group (Dorscht et al., 2009). However, A006, A118, and A500 were assigned to three distinct temperate phage clusters in this study, and phage PSA, though demonstrated genomic similarity to B025, remained a singleton due to its mosaic genome containing modules from both *Listeria* phage and phages infecting other *Firmicutes* (Dorscht et al., 2009). These findings underscore the importance of expanded genomic datasets for enhancing *Listeria* phage classification and advancing our understanding of their evolutionary relationships. It is first time to incorporates the most recent genome sequences of *Listeria* phages, as well as including a systematic analysis of the evolutionary diversity of three representative proteins, major capsid protein (MCP), receptor-binding protein (RBP), and endolysin in this study, which fills a critical gap in the classification of *Listeria* phage, and deepens our understanding of the distribution and diversity of these representative proteins across all defined clusters.

Based on a geographically broad collection of *Listeria* phage isolated from four continents, temperate phages exhibit greater genomic diversity than virulent phages in this study. Three proteins (MCP, RBP, and endolysin) play fundamental roles in phage structure, host recognition, and lysis, respectively. MCP, encoded by conserved structural genes, forms the icosahedral capsid that encapsulates the phage genome (Cucic et al., 2025). RBP has been shown to facilitate host recognition by specifically binding to bacterial surface receptors, most commonly wall teichoic acids (WTAs) in *Listeria monocytogenes* (Yang et al., 2024). Endolysins, encoded by phage genomes, are peptidoglycan hydrolases that degrade the bacterial cell wall to enable the release of progeny phages at the end of the lytic cycle (Yang et al., 2024; Chang et al., 2022; Gupta and Adhikari, 2022). In virulent phage clusters (Clusters 1 and 2), the major capsid protein (MCP), receptor-binding protein (RBP), and endolysin are conserved, reflecting strong selective pressure for efficient infection and host lysis. The observed differences between these clusters are likely attributable to host specificity or niche adaptation. Conversely, temperate phage clusters exhibit a heterogeneous distribution of these proteins, which probably attributable to weaker selective constraints and increased horizontal gene transfer (HGT) during the lysogenic cycle (Dion et al., 2020; Denes et al., 2014; Pedulla et al., 2003; Mmolawa et al., 2003; Casjens and Thuman-Commike, 2011; Luo et al., 2022; Moura de Sousa et al., 2021; Bhaya et al., 2025; Zhu et al., 2023). The cluster-specific analysis yielded several noteworthy patterns. MCP variation within Cluster 9 indicates adaptation to environmental or host-associated pressures. The strong correlation between RBP domain architecture and phage clusters supports the hypothesis of adaptive evolution toward distinct host receptors. Cluster 3 phages lacked identifiable RBP sequences, indicating the absence of canonical receptor-binding domains readily detectable by standard domain prediction tools. Previous bioinformatic and structural studies have shown that some phages lack apparent conventional RBP domains yet may still mediate host recognition through non-canonical or unannotated proteins, rather than classical tail fiber or tail spike RBPs (Boeckaerts et al., 2022; Matusiak et al., 2025). Furthermore, the substantial heterogeneity of endolysin domains observed in Clusters 5, 6, and 9 may underpin the ability to target a broader range of host cell walls. The extensive variation in endolysin domain composition highlights the complex strategies employed by *Listeria* phage to optimize host cell wall degradation, reflecting ecological and evolutionary diversification across clusters.

Cluster 1 comprises the largest group of *Listeria* phage with available genomic data, accounting for 44.33% of the total. These phages are predominantly broad-host-range virulent types, and several, including P100, have been evaluated for their effectiveness in phage biocontrol applications (Abbas et al., 2022; Carlton et al., 2005; Kim et al., 2008). Cluster 1 phages are considered to be ecologically advantaged based on genomic characteristics, and all of these belong to the genus *Pecentumvirus*, their genome sizes range from 131 to 139 kilobases, encoding 174–206 open reading frames, with a guanine-cytosine content of 35.96% ± 0.08%. All members of the species encode 10–17 tRNAs. Cluster 1 phages also encode four AMGs, exhibiting a clear signature of metabolic enhancement that likely supports efficient replication by strengthening host nucleotide metabolism and the metabolism of cofactors and vitamins. The major capsid protein (MCP), receptor-binding protein (RBP), and endolysin demonstrate significant conservation. Specifically, for Cluster 1 phages, the MCP belongs to Type C1 and contains the SU10 major capsid protein domain; the RBP is classified as Type R1 and contains the PHA01818 superfamily domain; and the endolysin belongs to Type E1, containing both an N-acetylmuramoyl-L-alanine amidase domain and a DUF3597 superfamily domain. These phages exhibit a broad geographical distribution across all four continents (North America, South America, Europe, and Asia), with diverse sources including food, food-related environments, and natural environments.

However, there are still some limitations in this study. The number of reported *Listeria* phage genomes remains limited. A significant proportion of phage genomes available on the NCBI database are devoid of detailed metadata regarding their isolation sources, thereby impeding the facilitation of comparative analyses across diverse environmental origins. Furthermore, their phenotype including the host range was not characterized for a significant proportion of phages across clusters. Despite the host range has been investigated in many cases, differences in host strain selection have been shown to reduce comparability between studies, thereby limiting insights into cluster-specific traits. Moreover, the identification of 23 putative RBPs was solely based on HHpred predictions without experimental validation, which may affect the accuracy of functional assignments. In cases where HHpred returned multiple candidates for a given phage, only a single top-ranking RBP was retained for downstream analyses. This strategy may underestimate RBP diversity and compromise the ability to fully resolve associations between RBP types and host range. The future research need to concentrate on the isolation of novel phages and the dissemination of comprehensive genome sequence and metadata, with a view to enhancing our understanding of *Listeria* phage diversity. Further studies should investigate the molecular mechanisms and application potential of phage-host interactions across clusters to better define their distinguishing features.

## 5 Conclusion

This study provides novel insights into classification and expands the understanding of phylogenetic diversity, evolutionary dynamics, and global distribution of of *Listeria* phage. Based on the complete *Listeria* phage genomes that are currently available, nine genomic clusters were defined, comprising two virulent and seven temperate clusters. Temperate phages demonstrate greater genomic diversity than virulent phages. All of the Cluster 1 phages belong to the genus *Pecentumvirus*, and are considered to be ecologically advantaged based on genomic characteristics. Three representative phage proteins (MCP, RBP and endolysin) were classified to nine, seven and eight distinct types, and conserved within virulent clusters and diversified within temperate clusters. The RBP of types R1, R2, R4, and R6 were linked to a broad host range and distributed across Clusters 1, 2, 7, and 8 phages. The molecular mechanisms and application potential of phage-host interactions deserve further investigation, which will facilitate more precise definition of *Listeria* phage cluster-specific biological traits.

## Data Availability

The genome assemblies generated in this study have been deposited in the National Microbiology Data Center (NMDC) under accession numbers NMDC60227322-NMDC60227337.

## Supplementary material

Supplemental Table S1 Summary of origin, genomic, and biological features of 97 *Listeria* phages. This table is provided as a separate Excel file attached to the submission.

Supplemental Figure S1 Comparison of the 16 newly isolated *Listeria* phages from three clusters with previously reported phages based on the viral proteomic tree A. displays eight newly isolated broad-host-range virulent phages (Phage39, Phage130, Phage144, Phage189, Phage201, Phage208, Phage211, and Phage222), together with *Listeria* phage vB-LmoM-SH3-3 and *Listeria* phage LMLPA3. B. shows four newly isolated broad-spectrum temperate phages (Phage263, Phage17, Phage33, and Phage19), along with *Listeria* phage A118 and *Listeria* phage 184. C. presents four newly isolated serotype 4-specific narrow-host-range phages (Phage62, Phage102, Phage225, and Phage251) together with *Listeria* phage HB17054.
