# Supplementary figures and images for: Genome-based taxonomic classification of Listeria phage and diversity analysis of major capsid protein, receptor-binding protein and endolysin
Source: Front Microbiol. 2026 Mar 4;17:1767683. doi: 10.3389/fmicb.2026.1767683 (PMC12996196; doi:10.3389/fmicb.2026.1767683)

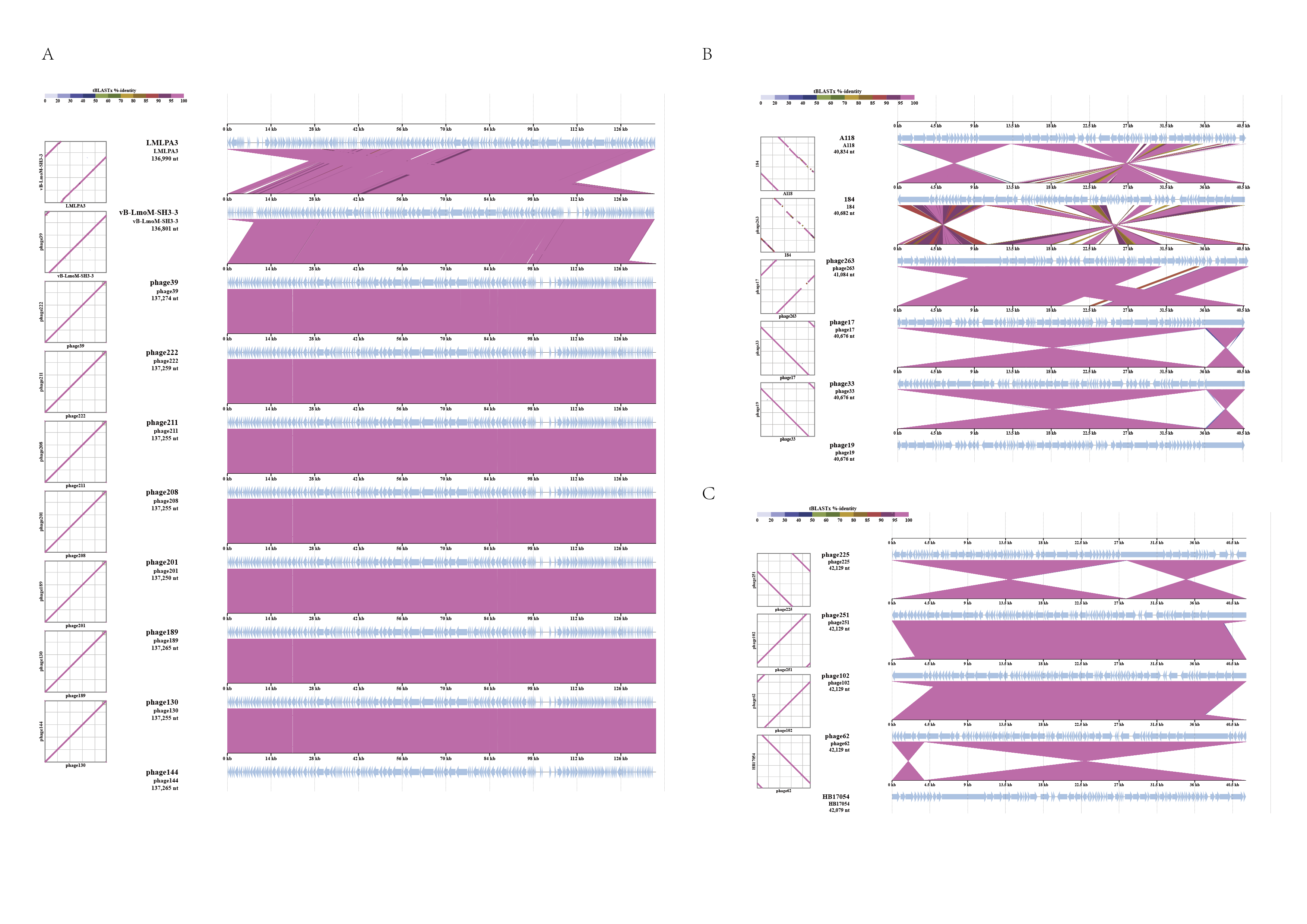

Supplement: Supplemental Figure S1 — Comparison of the 16 newly isolated Listeria phages from three clusters with previously reported phages based on the viral proteomic tree A. displays eight newly isolated broad-host-range virulent phages (Phage39, Phage130, Phage144, Phage189, Phage201, Phage208, Phage211, and Phage222), together with Listeria phage vB-LmoM-SH3-3 and Listeria phage LMLPA3. B. shows four newly isolated broad-spectrum temperate phages (Phage263, Phage17, Phage33, and Phage19), along with Listeria phage A118 and Listeria phage 184. C. presents four newly isolated serotype 4-specific narrow-host-range phages (Phage62, Phage102, Phage225, and Phage251) together with Listeria phage HB17054. [file Supplementary_file_2.tif]
